# Ceramides and phospholipids in plasma extracellular vesicles are associated with high risk of major cardiovascular events after carotid endarterectomy

**DOI:** 10.1038/s41598-022-09225-6

**Published:** 2022-04-01

**Authors:** Nathalie Timmerman, Farahnaz Waissi, Mirthe Dekker, Gert J. de Borst, Joelle van Bennekom, Robbert J. de Winter, Mika Hilvo, Antti Jylhä, Gerard Pasterkamp, Dominique P. V. de Kleijn, Reijo Laaksonen

**Affiliations:** 1 Department of Vascular Surgery (G04129), University Medical Centre Utrecht, Utrecht University, Heidelberglaan 100, 3584 CX Utrecht, The Netherlands; 2 Department of Cardiology, Amsterdam Cardiovascular Sciences, Academic Medical Center, Amsterdam UMC, Amsterdam, The Netherlands; 3 Zora Biosciences, Tietotie 2C, 02150 Espoo, Finland; 4 Laboratory of Clinical Chemistry and Hematology, Division Laboratories and Pharmacy, University Medical Centre Utrecht, Utrecht University, Utrecht, The Netherlands; 5 Finnish Cardiovascular Research Center Tampere, Tampere University, Tampere, Finland

**Keywords:** Prognostic markers, Atherosclerosis, Carotid artery disease

## Abstract

Ceramides and phosphatidylcholines (PCs) are bioactive lipids and lipid bilayer membrane components. Distinct ceramides/PCs (ratios) predict cardiovascular outcome in patients with coronary artery disease. Extracellular vesicles (EVs) are proposed biomarkers for cardiovascular disease and contain ceramides/PCs. Ceramides/PCs have not been studied in patients undergoing carotid endarterectomy (CEA) nor in EVs. We therefore investigated whether levels of ceramides/PCs in plasma and EVs are associated with postoperative risk of major adverse cardiovascular events (MACE) following CEA. In 873 patients undergoing CEA of the Athero-Express biobank, we quantitatively measured seven ceramides/PCs in preoperative blood samples: Cer(d18:1/16:0), Cer(d18:1/18:0), Cer(d18:1/24:0), Cer(d18:1/24:1), PC(14:0/22:6), PC(16:0/16:0) and PC(16:0/22:5) in plasma and two plasma EV-subfractions (LDL and TEX). We analyzed the association of ceramides, PCs and their predefined ratios with the three-year postoperative risk of MACE (including stroke, myocardial infarction and cardiovascular death). A total of 138 patients (16%) developed MACE during the three-year follow-up. In the LDL-EV subfraction, higher levels of Cer(d18:1/24:1) and Cer(d18:1/16:0)/PC(16:0/22:5) ratio were significantly associated with an increased risk of MACE (adjusted HR per SD [95% CI] 1.24 [1.01–1.53] and 1.26 [1.04–1.52], respectively). In the TEX-EV subfraction, three ratios Cer(d18:1/16:0)/Cer(d18:1/24:0), Cer(d18:1/18:0)/Cer(d18:1/24:0) and Cer(d18:1/24:1)/Cer(d18:1/24:0) were positively associated with MACE (adjusted HR per SD 1.34 [1.06–1.70], 1.24 [1.01–1.51] and 1.31 [1.08–1.58], respectively). In conclusion, distinct ceramides and PCs in plasma EVs determined in preoperative blood were independently associated with an increased 3-year risk of MACE after CEA. These lipids are therefore potential markers to identify high-risk CEA patients qualifying for secondary preventive add-on therapy.

## Introduction

Carotid endarterectomy (CEA) is a common effective treatment to lower the risk of future ipsilateral stroke in patients with a high degree asymptomatic or symptomatic extracranial carotid artery stenosis. Despite CEA, the residual risk for future cardiovascular (CV) events after CEA is still markedly high with approximately 20% in the three years after CEA^1^. CEA patients with high risk for secondary CV events qualify for more intensive medical treatment^2^, for instance by increasing the dose of statins or addition of PCSK-9 inhibitors. Also, anti-inflammatory drug therapies (such as colchicine), addition of anticoagulants or sodium-glucose cotransporter 2 (SGLT2) inhibitors are potential options that are currently under investigation^3–8^. These intensified treatments are often accompanied by high costs or detrimental side effects. Considering the varying risk of MACE across patients^9^, risk stratification tools that assist individualized secondary prevention are warranted. However, clinical prediction models including CV risk factors have poor predictive ability to discriminate between high and low risk CEA patients for future CV events^9,10^. Biomarkers may improve risk stratification models to identify patients at high risk for future CV events.

Previous lipidomic studies have identified ceramides and phosphatidylcholines (PCs) as promising biomarkers for cardiovascular disease^11–17^. Ceramides belong to sphingolipids consisting of a sphingosine backbone with an attached fatty acid. PCs are phospholipids that are characterized by a choline headgroup and two fatty acyl side chains. Both are main components of lipid bilayer membranes but also act as key signaling molecules^18^. Ceramides and PCs are suggested to affect atherogenic processes and CV risk factors such as diabetes and obesity^18,19^. In patients with established coronary artery disease (CAD), particular ceramides and PCs and their distinct ratios were predictive for CV death and future CV events^11–17^. Until date, no studies have evaluated the role of ceramides and PCs for future CV events in patients following CEA.

Plasma extracellular vesicles (EVs) are a novel source of biomarkers for cardiovascular disease^20^. EVs are lipid bilayer membrane microstructures that transfer biological information including lipids (e.g. ceramides and PCs), proteins and RNA over distance from cell to cell. EVs originate either from budding of the outer cell membrane or subcellular compartments hereby including parent-cell membrane lipids and enclosing cytosolic material that could affect remote cells and influence pathophysiological processes^20^. The biological information in EVs is derived from the cell of origin and can reflect its status. It is known that EV subsets vary in size and in their biological content^20,21^. Protein content in EVs have been associated with recurrent CV events^22^. However, the content of ceramides and PCs in plasma EVs in relation with CV events have not yet been studied.

We therefore aimed to investigate whether circulating levels of ceramides and PCs in plasma and in two plasma EV subsets (the LDL- and TEX subfraction) are associated with the postoperative risk of MACE in patients undergoing CEA.

## Methods

### Study participants

Study participants originated from the Athero-Express Biobank. This ongoing prospective biobank study includes consecutive patients undergoing CEA in two tertiary referral hospitals (University Medical Center Utrecht and St. Antonius Hospital Nieuwegein, The Netherlands). A comprehensive description of the study design has been published earlier^1,23^. All patients undergoing CEA were asked for study participation. Indications for CEA were adjudicated by a multidisciplinary vascular team and based on recommendation from the European Carotid Surgery Trial^24^ and the North American Symptomatic Carotid Endarterectomy Trial^25,26^ for symptomatic patients and Asymptomatic Carotid Surgery Trial ^27,28^ for asymptomatic patients. Patients included in the Athero-Express Biobank from April 1st, 2002 until December 31, 2016 were eligible for the current study. Inclusion criteria were availability of citrate blood plasma sample and follow-up data. Exclusion criteria were patients undergoing CEA for restenosis. All patients provided written informed consent. Ethical approval for study conduction was provided by the Medical Research Ethics Committee United (MEC-U) of St. Antonius Hospital Nieuwegein, The Netherlands on April 10, 2002 (TME/C01.18). Study conduction complied with the Declaration of Helsinki.

### Data collection

Baseline characteristics were collected by standardized questionnaires that were verified against medical records including medical history, medication use, cardiovascular risk factors and basic laboratory parameters. A preoperative blood sample was taken and stored in − 80 °C freezer until further use. The atherosclerotic plaque was freshly obtained during CEA and immediately transferred to the laboratory for further histological analyses.

### Follow-up

Patients underwent yearly follow-up for a total duration of 3 years after CEA through standardized questionnaires send by post inquiring whether a patient had experienced any CV event or had been admitted to a hospital in the past year. Follow-up questionnaires were cross-checked with hospital medical records. In case of no response to questionnaires or when additional information regarding a CV event was necessary, the general practitioner was consulted to provide additional follow-up information consisting of medical records and hospital discharge letters from institutions where the event had occurred. All information regarding potential CV events were reviewed by two independent researchers. In case of disagreement, a third expert (GJdB) was consulted.

### Study outcomes

The primary outcome of the current study was defined as the 3-year postoperative risk of major adverse CV events (MACE) including fatal- or nonfatal ischemic or hemorrhagic stroke, fatal- or nonfatal myocardial infarction (MI) and any CV death also including sudden cardiac death, fatal aneurysm rupture and fatal cardiac failure. The definition of MACE was in concordance with a previous study in coronary patients and the European Perioperative Clinical Outcome (EPCO) definition^12,29^. Secondary outcomes were histological atherosclerotic carotid plaque characteristics: content of macrophages, smooth muscle cells (SMCs), collagen, calcification, intraplaque hemorrhage (IPH), intraplaque vessels and lipid core size.

### Biomarker selection

Selection of ceramides and phosphatidylcholines (PCs) was based on previous biomarker discovery and validation studies in CAD patients^11–14^. Ratios of ceramide/ceramide or ceramide/PC were predefined based on proven associations with CV outcomes in CAD cohorts^11–14,16^.

### Measurement of ceramides/PCs in plasma and EV-subfractions

Levels of ceramides and PCs were measured in unfractionated plasma and in two subpopulations of plasma extracellular vesicles (EV) called the LDL-EV subfraction and TEX-EV subfraction. A detailed overview of the EV isolation procedure has been reported previously^21^ and is described in the “Supplemental Materials”. In brief, the LDL-EV subfraction was precipitated using Dextran Sulphate (DS, 0.05%, MP Biomedicals) and Manganese II Chloride (MnCl_2_, 0.05 M, Sigma-Aldrich), the TEX-EV fraction with Xtractt buffer (1:4, Cavadis BV). Magnetic dextran nanoparticles (nanomag^®^-D plain for the LDL-EV fraction and Nano-mag^®^-D PEG-OH for the TEX-EV fraction) were added and EVs were isolated with use of a bio-plex handheld magnet. The pellet, containing the EVs, was lysed with lysis buffer to free its content. Magnetic nanoparticles and debris were separated and removed from the pellet by centrifugation. Ceramides and PC concentrations were quantified in plasma and in the LDL-EV and TEX-EV subfractions using liquid chromatography-mass spectrometry (LC–MS, Sciex TripleQuad 5500 mass spectrometer coupled to Sciex MPX LC system). Final concentrations of ceramides and PCs were expressed in µM. Details of the LC–MS quantification analyses of ceramides and PCs are stated in the “Supplemental Materials”. EV characterization in plasma EV subfractions have been described in previous studies^21,30,31^. Previous Nanoparticle Tracking Analyzer (NTA) experiments showed the smallest EVs in the TEX-fraction (mean 84 nm) and relatively larger EVs in LDL-fraction (mean 101 nm)^21^. Additional density gradient analyses of EV subfractions confirmed the presence of ceramides and PCs in EVs (see “Supplemental Materials” for details; Figs. S2, S3 and Table S1).

### Histological atherosclerotic plaque characterization

Histological examination of the carotid atherosclerotic plaque was performed according to the standardized Athero-Express biobank protocol^1,23^. Details are described in the “Supplemental Materials”^1,23^. The carotid plaque was cross-sectionally cut into segments of 5 mm. The segment with the largest plaque volume was considered the culprit lesion was allocated to immunohistochemical analysis of collagen, smooth muscle cells (SMCs), macrophages, calcifications, intraplaque hemorrhage (IPH) and lipid core. Plaque characteristics were scored semi quantitatively as no/minor or moderate/heavy staining, except for IPH (scored as absent or present) and lipid core (size was visually estimated relative to the total plaque area and expressed as < 10%, 10–40%, > 40% of the total plaque area). In addition, SMCs, macrophages and intraplaque vessels were quantified by computerized analysis software (AnalySIS 3.2, Soft Imaging Systems GmbH, Munster, Germany). SMCs and macrophage infiltration were expressed as the percentage of positive staining of the total plaque area. CD34 positive intraplaque vessels were counted in three hotspots with highest vessel density and the average number per square millimeter was calculated, as described previously^32^.

### Statistical analyses

#### Associations with MACE

Continuous baseline characteristics and categorical baseline characteristics were respectively compared by Students t-test or Mann Whitney U test and Pearson’s Chi-squared test. Cholesterol and creatinine levels were logarithmically transformed because of skewness. The associations of ceramides and PCs (ratio) levels with the 3-year postoperative risk of MACE were analyzed by univariable, adjusted for LDL-C and HDL-C, and multivariable Cox proportional hazards models, similar to the previous CAD study^11^. To facilitate easy comparison with existing literature ceramides and PCs (ratios) concentrations were standardized to Z-scores^11,12^. Hazard ratios (HR) were expressed per one SD increase. Potential confounders for multivariable analyses were a priori selected based on available literature^10,11,14,16,33,34^; age, history of coronary artery disease (CAD) and/or peripheral artery disease (PAD), preprocedural cerebrovascular symptoms, current smoking, hypertension, diabetes, lipid-lowering drug use, triglycerides, total cholesterol, creatinine and contralateral carotid artery stenosis of 50–100%. Model reduction using Akaike information criterion in a stepwise backward regression resulted in the final model including age, history of CAD and/or PAOD, cerebrovascular symptoms, current smoking, LDL-C and HDL-C. Percentage of missing covariates was low (range 0.0–4.2%), see Supplemental Table S2. To gain further insight in the association of ceramides and PCs (ratios) and the occurrence of MACE during three-year follow-up, multivariable significant ceramides/PCs were visualized in quartiles by Kaplan–Meier graphs.

#### Predictive value for MACE

To explore the added predictive value of ceramide/PCs (ratios) for MACE on top of clinical risk factors, model performance was assessed. Bootstrapping techniques were used for internal validation in order to control for optimism and overfitting. Calibration was assessed by visual inspection of the calibration plots. Discriminative performance was assessed by calculating the C-index and the Integrated Discrimination Improvement (IDI)^35^.

#### Association with plaque characteristics

To unravel potential underlying biological mechanisms, the association of multivariable significant ceramides and PCs (ratios) with histological atherosclerotic plaque characteristics were investigated by univariable and multivariable logistic or linear regression. Age, sex, LDL-C, HDL-C, triglycerides and total cholesterol were added as potential confounders for multivariable analyses of plaque characteristics^34,36^. All p-values resulted from two-tailed hypothesis testing. A *p*-value < 0.05 indicated statistically significance. Analyses were performed in R statistical software version 3.6.2 (R Foundation for Statistical Computing, Vienna, Austria; https://www.r-project.org/).

## Results

### Study population

**Figure 1:**
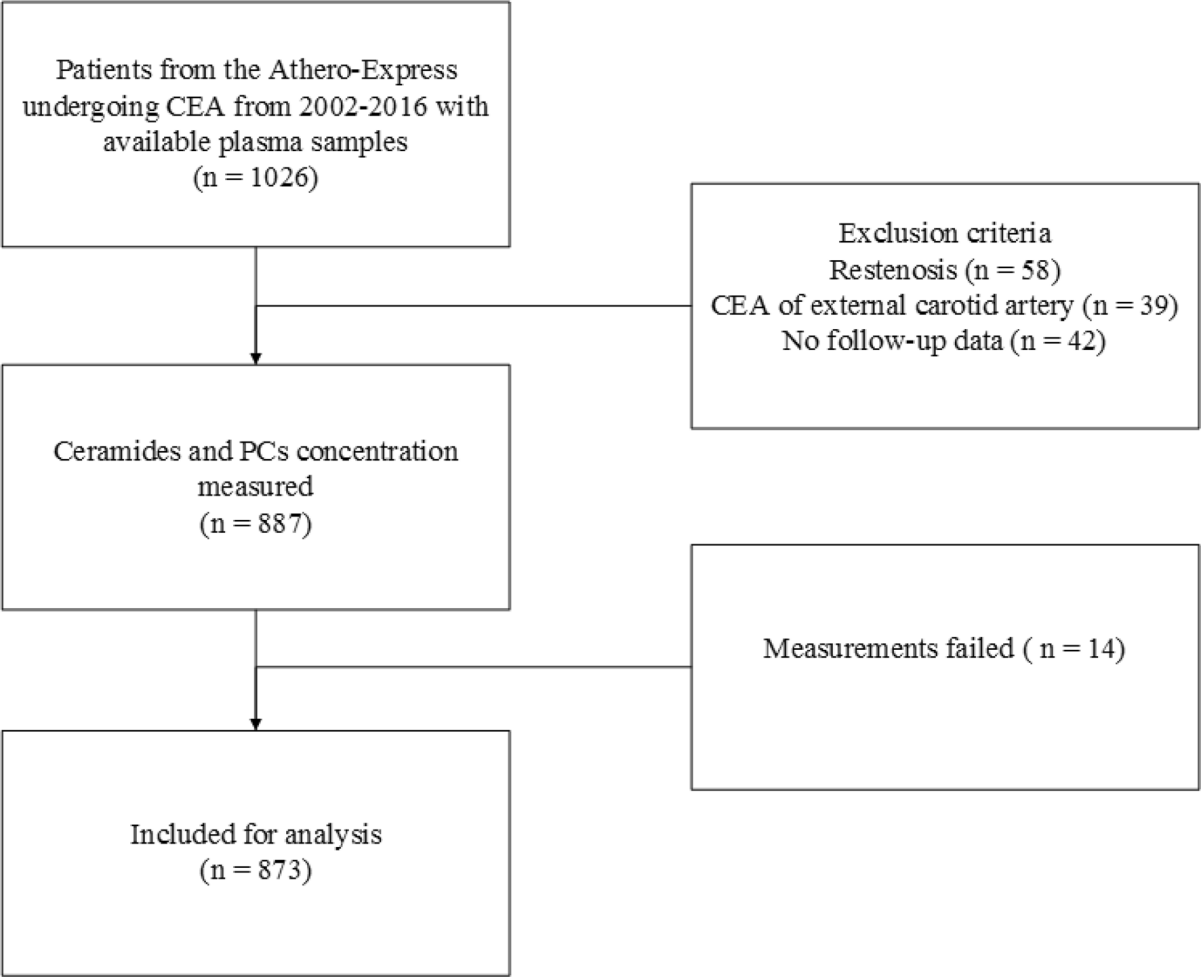
Flowchart of included patients in the present study.

A total of 887 patients were included in the current study (Fig. 1). Measurement of ceramides and PCs failed in 14 patients leaving 873 patients for analyses. Patient characteristics are summarized in Table 1. The overall mean age was 69 years, 70% were men and most were operated for high degree (70–99%) symptomatic carotid stenosis. The prevalence of CV risk factors, history of CAD and PAD were high, exemplified by the high frequency of antiplatelet and lipid lowering drug use at study inclusion. Patients that experienced MACE after three years of follow up were significantly older, were more often diabetic, more often had a history of coronary or peripheral artery disease, had higher creatinine levels and had lower HDL levels at baseline than patients that remain free from MACE.

**Table 1:** Baseline characteristics.

	No MACE (n = 735)	MACE (n = 138)	*p-*value
**Demographics**			
Age, mean (SD)	68.8 (9.2)	72.1 (9.0)	**< 0.001**
Male	508 (69.1)	103 (74.6)	0.231
Ipsilateral carotid artery stenosis degree			
50–70%	62 (8.7)	6 (4.5)	
70–99%	654 (91.3)	128 (95.5)	0.119
**Risk factors**			
Contralateral carotid artery stenosis of 50–100%	292 (44.0)	66 (51.6)	0.138
BMI, mean (SD)	26.29 (3.9)	26.71 (3.9)	0.250
Hypertension	609 (82.9)	118 (86.1)	0.412
Creatinine, median [IQR]	87.0 [75.0, 104.0]	95.0 [81.0, 114.0]	**0.001**
GFR, mL/min/1.73 m^2^, mean (SD)	74.1 (20.3)	67.7 (21.3)	**0.001**
Current smoking	235 (32.3)	52 (38.2)	0.210
Diabetes	156 (21.2)	43 (31.2)	**0.015**
Hypercholesterolemia	467 (69.6)	80 (67.8)	0.777
Triglycerides levels, mmol/L, median [IQR]	1.5 [1.1, 2.1]	1.5 [1.1, 2.0]	0.803
LDL levels, mmol/L, median [IQR]	2.3 [1.8, 3.0]	2.3 [1.8, 3.0]	0.496
HDL levels, mmol/L, median [IQR]	1.1 [0.9, 1.3]	0.9 [0.8, 1.1]	**< 0.001**
Total cholesterol levels, mmol/L, median [IQR]	4.2 [3.5, 5.2]	4.1 [3.5, 4.9]	0.125
**Medical history**			
Cerebrovascular symptoms			0.206
asymptomatic	306 (41.6)	45 (32.6)	
ocular	78 (10.6)	16 (11.6)	
TIA	204 (27.7)	39 (28.2)	
stroke	147 (20.0)	38 (27.5)	
History of stroke or TIA	556 (75.6)	110 (79.7)	0.357
History of CAD or PAD			**0.005**
no history of CAD and PAD	429 (58.7)	65 (47.1)	
history of CAD or PAD	249 (34.1)	53 (38.4)	
history of CAD and PAD	53 (7.3)	20 (14.5)	
**Drug therapy**			
Anticoagulants	70 (9.5)	20 (14.6)	0.102
Antiplatelets	648 (88.4)	118 (86.8)	0.690
Lipid lowering drugs	591 (80.5)	102 (74.5)	0.133

Values are displayed as frequency, n (%) unless otherwise indicated. Values in bold are *p-*value < 0.05. Categorical baseline characteristics were compared by Pearson’s Chi-squared test. Continuous baseline characteristics were compared by Students t-test or Mann Whitney U based on the distribution of the data.

*SD *standard deviation, *IQR *interquartile range, *BMI* body mass index, *CAD* coronary artery disease, *PAD *peripheral artery disease, *GFR* estimated glomerular filtration rate calculated by MDRD equation, *TIA* transient ischemic attack, *LDL *low-density lipoprotein, *HDL* high-density lipoprotein.

Hypertension, diabetes and hypercholesterolemia were defined as diagnosed by a medical doctor or use of specific medication. Antiplatelet drug comprises the use of aspirin, dipyridamole or any ADP-inhibitor. Lipid lowering drug use comprises the use of any lipid lowering drug. History of stroke or TIA includes ipsilateral or contralateral stroke or TIA.

Cerebrovascular symptoms refer to the ipsilateral symptoms on which the indication for CEA was based. Asymptomatic is defined as no experience of ipsilateral cerebrovascular symptoms in the six months prior to CEA.

During a median postoperative follow-up duration of 3.0 years [IQR 2.2–3.0], 138 patients (15.8%) experienced MACE consisting of 74 (8.5%) fatal and non-fatal stroke, 44 (5%) fatal and non-fatal MI and 20 (2.3%) CV death due to other causes. Patients that experienced MACE were at inclusion significantly older, more often had diabetes, had lower HDL-C levels, higher creatinine levels and were more likely to have a history of CAD and/or PAD compared to patients who remained free from MACE (Table 1). Availability of ceramide and PC measurements, as well as absolute concentrations compared between patients that experienced MACE and those that did not, are shown in Supplemental Table S3.

### Associations with the 3-year postoperative risk of MACE

Univariable and multivariable associations of ceramides and PCs, measured in plasma and EV subfractions, with the 3-year risk of MACE are depicted in Table 2. After correction for all confounders (LDL-C, HDL-C, age, history of CAD and/or PAD, cerebrovascular symptoms and current smoking), one ceramide and four ceramide ratios remained significantly associated with MACE in the LDL-EV subfraction as well as in the TEX-EV subfraction (Table 2).

**Table 2:** Associations of ceramides, PCs and predefined ratios with the 3-year postoperative risk of MACE after CEA.

Biomarker	Univariable		Adjusted for LDL-C and HDL-C		Multivariable	
	HR (95% CI)	p-value	HR (95% CI)	p-value	HR (95% CI)	p-value
**Cer(d18:1/16:0)**						
Plasma	1.00 (0.81–1.22)	0.961	1.07 (0.86–1.34)	0.538	0.98 (0.78–1.24)	0.878
LDL-EV	1.12 (0.93–1.36)	0.243	1.31 (1.06–1.62)	**0.012**	1.22 (0.99–1.51)	0.067
TEX-EV	0.96 (0.60–1.54)	0.861	0.92 (0.57–1.50)	0.751	0.94 (0.56–1.58)	0.826
**Cer(d18:1/18:0)**						
Plasma	1.04 (0.85–1.29)	0.680	1.07 (0.87–1.32)	0.530	1.00 (0.80–1.25)	0.993
LDL-EV	1.05 (0.87–1.29)	0.601	1.13 (0.92–1.37)	0.236	1.06 (0.86–1.31)	0.559
TEX-EV	0.97 (0.73–1.29)	0.855	0.93 (0.70–1.24)	0.632	0.93 (0.68–1.27)	0.645
**Cer(d18:1/24:0)**						
Plasma	0.79 (0.64–0.96)	**0.018**	0.80 (0.64–1.00)	**0.047**	0.85 (0.68–1.06)	0.158
LDL-EV	0.88 (0.72–1.06)	0.168	0.96 (0.79–1.18)	0.725	1.05 (0.85–1.28)	0.663
TEX-EV	0.63 (0.46–0.86)	**0.004**	0.63 (0.45–0.88)	**0.007**	0.72 (0.52–1.01)	0.056
**Cer(d18:1/24:1)**						
Plasma	1.01 (0.83–1.22)	0.949	1.05 (0.86–1.29)	0.643	0.95 (0.77–1.19)	0.661
LDL-EV	1.19 (0.98–1.44)	0.078	1.34 (1.09–1.64)	**0.005**	1.24 (1.01–1.53)	**0.040**
TEX-EV	0.98 (0.75–1.27)	0.855	0.95 (0.72–1.25)	0.721	0.93 (0.70–1.24)	0.627
**PC(14:0/22:6)**						
Plasma	0.78 (0.63–0.97)	**0.026**	0.83 (0.66–1.05)	0.115	0.88 (0.70–1.10)	0.250
LDL-EV	0.83 (0.68–1.02)	0.084	0.90 (0.72–1.11)	0.320	0.93 (0.75–1.16)	0.520
TEX-EV	0.82 (0.65–1.02)	0.077	0.85 (0.68–1.08)	0.182	0.89 (0.71–1.12)	0.323
**PC(16:0/16:0)**						
Plasma	0.99 (0.82–1.20)	0.928	1.15 (0.93–1.42)	0.195	1.11(0.88–1.39)	0.383
LDL-EV	1.06 (0.87–1.29)	0.593	1.14 (0.92–1.41)	0.225	1.18 (0.95–1.47)	0.136
TEX-EV	0.87 (0.71–1.07)	0.191	0.89 (0.73–1.09)	0.255	0.94 (0.76–1.15)	0.537
**PC(16:0/22:5)**						
Plasma	0.82 (0.67–1.00)	0.052	0.90 (0.73–1.11)	0.330	0.93 (0.75–1.14)	0.461
LDL-EV	0.87 (0.72–1.06)	0.168	0.97 (0.79–1.19)	0.750	0.97 (0.79–1.19)	0.761
TEX-EV	0.75 (0.60–0.94)	**0.014**	0.80 (0.63–1.00)	0.054	0.83 (0.66–1.06)	0.131
**Cer(d18:1/16:0)/Cer(d18:1/24:0)**						
Plasma	1.28 (1.03–1.58)	**0.024**	1.28 (1.03–1.59)	**0.029**	1.10 (0.87–1.38)	0.428
LDL-EV	1.19 (0.97–1.46)	0.089	1.22 (0.99–1.50)	0.061	1.07 (0.85–1.34)	0.559
TEX-EV	1.61 (1.30–2.00)	**< 0.001**	1.57 (1.26–1.96)	**< 0.001**	1.34 (1.06–1.70)	**0.016**
**Cer(d18:1/18:0)/Cer(d18:1/24:0)**						
Plasma	1.25 (1.01–1.53)	**0.038**	1.20 (0.97–1.48)	0.086	1.09 (0.88–1.35)	0.439
LDL-EV	1.02 (0.83–1.26)	0.830	1.00 (0.81–1.24)	0.983	0.90 (0.73–1.13)	0.375
TEX-EV	1.42 (1.17–1.72)	**< 0.001**	1.35 (1.11–1.65)	**0.002**	1.24 (1.01–1.51)	**0.042**
**Cer(d18:1/24:1)/Cer(d18:1/24:0)**						
Plasma	1.26 (1.04–1.54)	**0.020**	1.23 (1.01–1.50)	**0.041**	1.08 (0.87–1.34)	0.476
LDL-EV	1.21 (1.00–1.46)	0.051	1.18 (0.98–1.44)	0.083	1.07 (0.87–1.31)	0.546
TEX-EV	1.51 (1.27–1.79)	**< 0.001**	1.43 (1.20–1.70)	**< 0.001**	1.31 (1.08–1.58)	**0.005**
**Cer(d18:1/18:0)/Cer(d18:1/16:0)**						
Plasma	1.00 (0.84–1.21)	0.959	0.97 (0.80–1.16)	0.714	0.96 (0.79–1.16)	0.654
LDL-EV	0.95 (0.79–1.14)	0.583	0.93 (0.77–1.12)	0.446	0.90 (0.74–1.09)	0.265
TEX-EV	0.96 (0.80–1.14)	0.613	0.92 (0.77–1.10)	0.354	0.93 (0.77–1.11)	0.412
**Cer(d18:1/16:0)/PC(16:0/22:5)**						
Plasma	1.40 (1.15–1.70)	**0.001**	1.36 (1.11–1.66)	**0.003**	1.22 (0.99–1.51)	0.060
LDL-EV	1.40 (1.16–1.69)	**< 0.001**	1.39 (1.15–1.67)	**0.001**	1.26 (1.04–1.52)	**0.016**
TEX-EV	1.58 (1.16–2.16)	**0.003**	1.39 (1.01–1.92)	**0.046**	1.28 (0.91–1.80)	0.161
**Cer(d18:1/18:0)/PC(14:0/22:6)**						
Plasma	1.30 (1.03–1.63)	**0.030**	1.23 (0.97–1.56)	0.087	1.09 (0.86–1.40)	0.473
LDL-EV	4.06 (0.86–19.1)	0.077	3.36 (0.70–16.0)	0.129	1.88 (0.35–10.1)	0.463
TEX-EV	1.00 (0.53–1.89)	0.993	0.80 (0.41–1.56)	0.513	0.81 (0.37–1.74)	0.583

Cox-regression analyses were performed to assess the association of ceramides, PCs and ratios with the 3-year postoperative risk of MACE. Cox regression analyses were performed in three ways: univariable, adjusted for LDL-C and HDL-C and multivariable corrected for LDL-C and HDL-C, age, history of CAD, history of peripheral artery disease, cerebrovascular symptoms and current smoking. HR indicates the hazard ratio for the 3-year postoperative risk of MACE per one standard deviation increase in concentration of ceramides, PCs or ratios, either in plasma or plasma extracellular vesicles (EVs). Values in bold indicate p < 0.05.

*CI* confidence interval, *Cer* ceramide, *PC* phosphatidylcholine, *LDL-EV* the LDL-EV subfraction, *TEX-EV* the TEX-EV subfraction.

In the LDL-EV subfraction, higher levels of Cer(d18:1/24:1) were independently associated with a higher 3-year postoperative risk of MACE with adjusted HR 1.24 per SD, 95% CI 1.01–1.53 (*p* = 0.040). A higher ratio of Cer(d18:1/16:0)/PC(16:0/22:5) in the LDL-EV subfraction was independently associated with a higher 3-year risk of MACE with adjusted HR of 1.26 per SD, 95% CI 1.04–1.52 (*p* = 0.016).

In the TEX-EV subfraction, three ceramide ratios were positively associated with the 3-year risk of MACE, namely Cer(d18:1/16:0)/Cer(d18:1/24:0) with adjusted HR 1.34 per SD, 95% CI 1.06–1.70 (*p* = 0.016), Cer(d18:1/18:0)/Cer(d18:1/24:0) with adjusted HR 1.24 per SD, 95% CI, 1.01–1.51 (*p* = 0.042) and Cer(d18:1/24:1)/Cer(d18:1/24:0) with adjusted HR 1.31 per SD, 95% CI 1.08–1.58 (*p* = 0.005).

In plasma, multiple ceramide ratios were significantly associated with MACE in univariable analyses, however, these became insignificant after correction for confounders.

As statins are known to modify ceramide/PC levels^14^, sub analyses with addition of statin use to multivariable models were performed (Supplemental Table S4). All the lipids or lipid ratios in the LDL-EV and two in the TEX-EV subfractions remained significantly associated with MACE after CEA, but Cer(d18:1/18:0)/Cer(d18:1/24:0) in TEX-EV subfraction became borderline non-significant (Supplemental Table S4).

**Figure 2:**
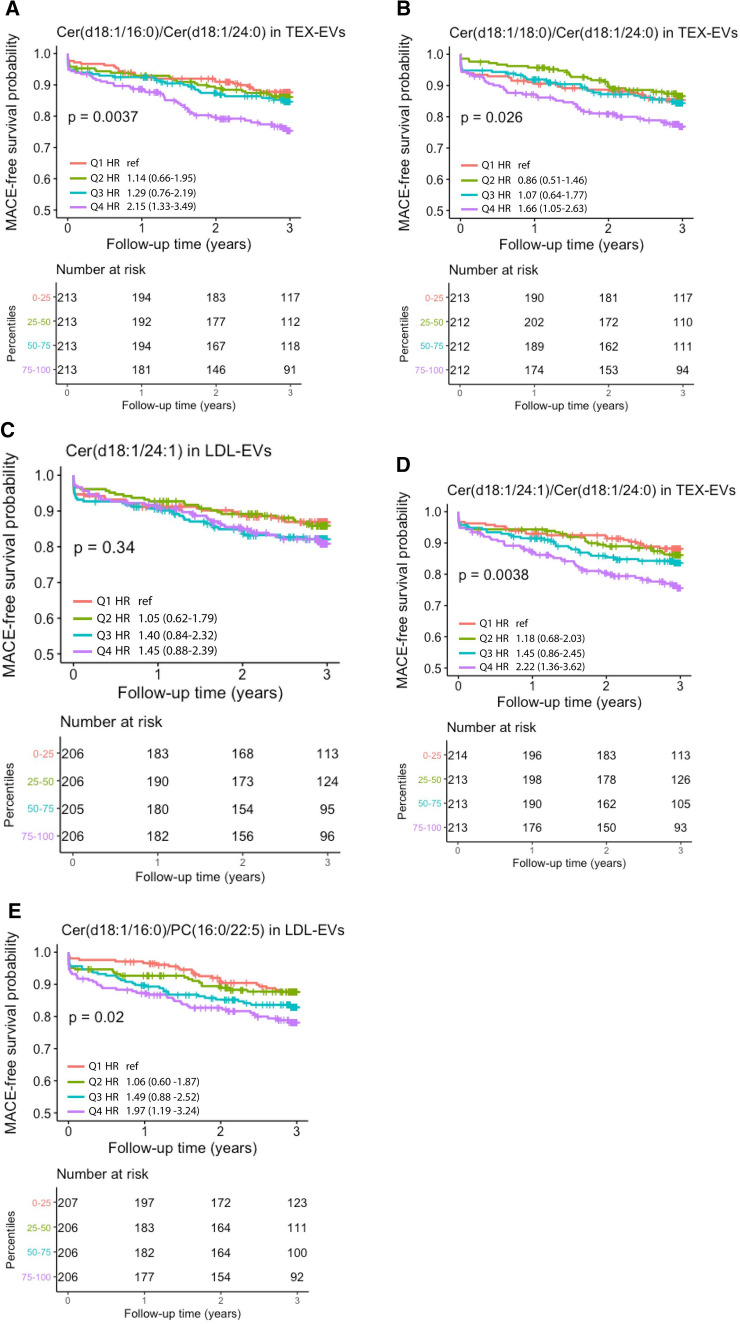
(**A**)–(**E**). Kaplan–Meier estimates of major adverse cardiovascular event (MACE) for quartiles of multivariable significant associated ceramides/PC ratios. The p-value indicates the overall comparison of the MACE-free survival across quartile levels by the log-rank test. The hazard ratios in the legends indicate the univariable quartile-specific hazard relative to the first quartile.

In order to gain further insight in the association of ceramide/PC ratios with MACE, we analyzed quartile levels by Kaplan–Meier plots and Cox-regression analyses (Fig. 2A–E). Non-linearity was found for the ratios of Cer(d18:1/16:0)/Cer(d18:1/24:0) and Cer(d18:1/18:0)/Cer(d18:1/24:0) in the TEX-EV fraction where patients in the 4th quartile were at relatively high risk (respective HR for patients in the 4th quartile versus those in the 1st quartile were 2.15, 95% CI 1.33–3.49 and 1.66, 95% CI 1.05–2.63) (Fig. 2A,B). The association of Cer(d18:1/24:1) in the LDL-EV fraction with MACE appeared to be dichotomous (Fig. 2C). Patients with levels above the median had a HR of 1.44, 95% CI 1.01–2.06, compared to patients with levels below the median. The 3rd and 4th quartiles of the Cer(d18:1/24:1)/Cer(d18:1/24:0) ratio in the TEX-EV subfraction and Cer(d18:1/16:0)/PC(16:0/22:5) ratio in the LDL-EV subfraction seemed to be more linearly associated with increased risk of MACE (Fig. 2D,E).

### Predictive value on top of clinical risk factors

Possible added predictive value of multivariable significant ceramides/PCs on top of clinical risk factors was evaluated using stepwise backward regression modelling. Based on likelihood estimates, addition of Cer(d18:1/24:1)/Cer(d18:1/24:0) ratio in the TEX-EV fraction improved the clinical model (including age, history of CAD and/or PAD, cerebrovascular symptoms, current smoking, LDL-C and HDL-C). Calibration plots showed improved calibration with the ceramide ratio (Supplemental Fig. S4). The C-statistic did not improve significantly (C-statistic of the clinical model was 0.67 and for the biomarker model with the ceramide ratio 0.68, p = 0.445 for comparison). Addition of the ceramide ratio to the clinical model resulted in a significant IDI of 0.03 (0.008–0.053), p = 0.008.

### Associations with histological atherosclerotic plaque characteristics

Having established an association of ceramide/PC EV-levels with MACE and considering that EVs probably reflect the status of the cell of origin, we investigated the association of the multivariable significant ceramides and PCs with histological plaque characteristics that are considered as key markers for plaque vulnerability. This might unravel potential underlying mechanisms how these lipid ratios contribute to cardiovascular event risk. Increased ratios of Cer(d18:1/18:0)/Cer(d18:1/24:0) and Cer(d18:1/24:1)/Cer(d18:1/24:0) in the TEX-EV fraction were inversely associated with the amount of SMCs in the plaque (respective adjusted odds ratio (OR) 0.76 for moderate/heavy SMC staining, 95% CI 0.61–0.95, *p* = 0.015) and adjusted beta of -0.26 for the percentage of SMCs staining, 95% CI − 0.47 to − 0.04, *p* = 0.020) (Table 3). An increased ratio of Cer(d18:1/16:0)/ PC(16:0/22:5) in the LDL-EV subfraction was positively associated with more macrophage infiltration (beta 0.16, 0.05–0.28, *p* = 0.007). Neither Cer(d18:1/24:1) in the LDL-EV fraction nor the ratio Cer(d18:1/16:0)/Cer(d18:1/24:0) in TEX-EV subfraction were associated with histological plaque characteristics.

**Table 3:** Association of ceramides, PCs and ratios with histological atherosclerotic carotid plaque characteristics.

Binary plaque characteristics	Cer(d18:1/24:1) in LDL-EV subfraction			Cer(d18:1/16:0)/PC(16:0/22:5) in LDL-EV subfraction		Cer(d18:1/16:0)/Cer(d18:1/24:0) in TEX-EV subfraction		Cer(d18:1/18:0)/Cer(d18:1/24:0) in TEX-EV subfraction		Cer(d18:1/24:1)/Cer(d18:1/24:0) in TEX-EV subfraction	
		OR (95% CI)	*p-*value	OR (95% CI)	*p-*value	OR (95% CI)	*p-*value	OR (95% CI)	*p-*value	OR (95% CI)	*p-*value
Calcification (moderate/heavy staining)	**UV**	0.93 (0.78–1.11)	0.440	0.96 (0.81–1.14)	0.633	0.95 (0.76–1.19)	0.659	0.92 (0.76–1.12)	0.421	0.85 (0.72–1.02)	0.076
	**MV**	0.85 (0.71–1.03)	0.102	0.93 (0.78–1.11)	0.415	0.90 (0.70–1.15)	0.398	0.92 (0.75–1.12)	0.394	0.84 (0.70–1.02)	0.073
Collagen (moderate/heavy staining)	**UV**	1.12 (0.91–1.39)	0.273	1.14 (0.92–1.40)	0.231	1.12 (0.85–1.47)	0.426	0.99 (0.79–1.24)	0.941	1.06 (0.86–1.30)	0.570
	**MV**	1.11 (0.89–1.38)	0.374	1.10 (0.89–1.36)	0.377	1.07 (0.80–1.44)	0.630	1.00 (0.79–1.26)	0.975	1.06 (0.85–1.32)	0.595
SMC (moderate/heavy staining)	**UV**	0.98 (0.81–1.19)	0.841	0.95 (0.79–1.15)	0.606	0.84 (0.66–1.07)	0.148	0.78 (0.63–0.95)	**0.014**	0.95 (0.79–1.14)	0.580
	**MV**	1.01 (0.82–1.24)	0.951	0.96 (0.79–1.16)	0.661	0.86 (0.66–1.12)	0.269	0.76 (0.61–0.95)	**0.015**	1.01 (0.83–1.24)	0.897
Presence of IPH	**UV**	1.20 (1.00–1.43)	0.053	1.17 (0.98–1.40)	0.083	1.03 (0.81–1.29)	0.834	0.96 (0.79–1.16)	0.665	1.02 (0.86–1.22)	0.813
	**MV**	1.18 (0.97–1.43)	0.108	1.10 (0.91–1.32)	0.335	0.97 (0.75–1.25)	0.803	0.96 (0.78–1.18)	0.678	1.00 (0.83–1.20)	0.974
Macrophages (moderate/heavy staining)	**UV**	0.93 (0.78–1.11)	0.438	1.10 (0.93–1.31)	0.276	0.92 (0.73–1.16)	0.472	0.91 (0.75–1.10)	0.327	0.91 (0.76–1.08)	0.282
	**MV**	0.87 (0.72–1.05)	0.136	1.10 (0.92–1.32)	0.290	0.98 (0.76–1.25)	0.865	0.96 (0.78–1.18)	0.704	0.93 (0.77–1.12)	0.445
Presence of lipid core > 40%	**UV**	0.93 (0.76–1.13)	0.468	1.11 (0.91–1.34)	0.297	0.96 (0.74–1.24)	0.765	0.95 (0.76–1.18)	0.618	0.94 (0.77–1.14)	0.527
	**MV**	0.84 (0.67–1.04)	0.101	1.08 (0.88–1.32)	0.475	1.03 (0.77–1.37)	0.832	1.06 (0.84–1.34)	0.624	0.96 (0.78–1.19)	0.736
Presence of lipid core > 10%	**UV**	1.07 (0.88–1.30)	0.500	1.04 (0.86–1.26)	0.675	0.99 (0.77–1.26)	0.918	0.99 (0.80–1.22)	0.916	1.01 (0.83–1.21)	0.956
	**MV**	1.01 (0.82–1.25)	0.908	0.98 (0.80–1.19)	0.809	0.98 (0.75–1.28)	0.884	1.04 (0.83–1.30)	0.752	0.99 (0.81–1.21)	0.912

Continuous plaque characteristics		Beta (95% CI)	*p-*value	Beta (95% CI)	*p-*value	Beta (95% CI)	*p-*value	Beta (95% CI)	*p-*value	Beta (95% CI)	*p-*value
Macrophages, % positive staining/total plaque	**UV**	− 0.03 (− 0.14–0.09)	0.658	0.14 (0.02–0.25)	**0.019**	0.04 (− 0.11–0.19)	0.607	− 0.05 (− 0.18–0.08)	0.435	− 0.06 (− 0.17–0.06)	0.350
	**MV**	− 0.05 (− 0.17–0.07)	0.442	0.16 (0.05–0.28)	**0.007**	0.12 (− 0.04–0.28)	0.140	− 0.03 (− 0.16–0.10)	0.680	− 0.03 (− 0.15–0.09)	0.652
SMCs, % positive staining/total plaque	**UV**	− 0.12 (− 0.32–0.09)	0.271	0.03 (− 0.17–0.24)	0.736	− 0.15 (− 0.42–0.12)	0.278	− 0.12 (− 0.35–0.11)	0.296	− 0.28 (− 0.49 to − 0.08)	**0.008**
	**MV**	− 0.10 (− 0.31–0.12)	0.384	0.10 (− 0.11–0.30)	0.351	− 0.08 (− 0.36–0.21)	0.599	− 0.14 (− 0.38–0.09)	0.234	− 0.26 (− 0.47 to − 0.04)	**0.020**
Intraplaque vessels, mean number per hotspot	**UV**	0.20 (− 0.40–0.79)	0.520	0.14 (− 0.45–0.73)	0.644	0.38 (− 0.44–1.19)	0.366	− 0.43 (− 1.10–0.24)	0.207	0.05 (− 0.58–0.67)	0.882
	**MV**	0.05 (− 0.58–0.68)	0.881	0.12 (− 0.50–0.73)	0.705	0.45 (− 0.43–1.32)	0.320	− 0.39 (− 1.09–0.32)	0.285	0.11 (− 0.56–0.77)	0.753

Associations of ceramides/PCs with binary plaque characteristics were analyzed by logistic regression. Associations with continuous plaque characteristics were analyzed by linear regression.

OR and betas are calculated per one standard deviation increase in concentration of ceramides and ceramides/PCs ratios.

*UV* univariable model; *MV* the multivariable model corrected for age, sex, LDL-cholesterol, HDL-cholesterol, triglycerides and total cholesterol levels.

Values in bold indicate p < 0.05. *CI* confidence interval, *Cer* ceramides, *PC* phosphatidylcholine, *SMC* smooth muscle cells, *IPH* intraplaque hemorrhage.

## Discussion

Despite CEA, there remains a high residual risk for future CV events in these patients after surgery. Early identification of these high-risk CEA patients would allow early initiation of add-on therapy to reduce cardiovascular event risk. In this study, we examined the association of circulating molecular lipids, previously associated with CV outcome in CAD patients, with future CV events in patients undergoing CEA. In plasma EVs, elevated levels of Cer(d18:1/24:1) and the ratios Cer(d18:1/16:0)/Cer(d18:1/24:0), Cer(d18:1/18:0)/Cer(d18:1/24:0), Cer(d18:1/24:1)/Cer(d18:1/24:0) and Cer(d18:1/16:0)/PC(16:0/22:5) were associated with elevated risk of MACE during the 3 years follow-up independently of conventional cardiovascular risk factors. EV-derived ceramides/PCs ratios may be considered as biomarkers for high residual CV risk.

Previous studies investigated ceramides/PCs only in plasma and did show associations of particular ceramides/PCs (ratios) with an increased risk of CV death and future CV events in patients with acute coronary syndrome (ACS) or stable CAD^11–13,17^. In the general population, elevated plasma levels of these specific ceramides were associated with both primary as well as recurrent MACE^16^. Although we only found associations of these lipid ratios in plasma EVs subsets in a CEA population, our results further contribute to the concept of ceramides and PCs as markers for future CV events. Specifically, Cer(d18:1/24:1)/Cer(d18:1/24:0) in TEX-EVs may be a promising biomarker to improve risk stratification for MACE. If confirmed in external validation studies as well as impact studies, this biomarker may be valuable in selecting patients at high risk for MACE that may benefit from intensified medical treatment.

This is the first study that examined levels of ceramides and PCs in plasma EVs whereas previous studies only investigated unfractionated plasma^11–13,17^. Although univariable analysis showed significant associations of ceramides and PCs in both unfractionated plasma and plasma EVs, in multivariable analyses only the associations in plasma EVs remained significant. EV subpopulations differ in vesicle size and it is known that the biological composition and clearance mechanisms vary across subpopulations^20^. Since unfractionated plasma contains all EV subpopulations next to other particles and complexes containing ceramides and PCs, differences in biological information between subpopulations might be masked and remain unnoticed. Therefore, the signal to noise ratio will probably be better for lipid levels in EV subpopulations than in unfractionated plasma. Our results suggest that lipid ratios in EVs may therefore be more potent biomarkers for MACE than in unfractionated plasma.

In general, the ratios of ceramides or ceramides/PCs species were more strongly associated with MACE than the individual lipid species, which is in line with prior studies in CAD patients^11–13^. Very long chain ceramides are produced by the Ceramide Synthase enzyme isotype 2 (CerS2; C24:0 or 24:1), while long chain (C16:0) are produced by other isoforms, CerS6 and CerS5^37^. CerS2 haplo-insufficiency, however leads to compensatory increase of C16:0 ceramides showing that, despite they are produced by different enzymes, changes in ceramide ratios are more pronounced when ceramide pathways are changing^37,38^. For this, ratios of different ceramides and PCs species may serve as a better reflection of the complex ongoing metabolic pathways and CV disease risk than individual ceramide and PC species.

Ceramides and PCs are related to CV events and modification of lipid profiles by medical or dietary interventions aiming to reduce cardiovascular event risk has increasingly gaining attention^34,39–42^. In patients, statins and PCSK-9 inhibitors reduced high-risk ceramide and PC levels^14,34^. Interestingly, PCSK-9 inhibition by loss-of-function mutation lowered relatively more high-risk ceramide levels than LDL-C levels, suggesting that PCSK-9 inhibitors not solely act via reducing LDL-C but also through profound changes in the lipid metabolism^14^. In patients with metabolic syndrome, treatment with pioglitazone (an antidiabetic drug) reduced high-risk ceramide levels and concomitantly enhanced insulin sensitivity^39^. A post-hoc analyses indicated that in patients with elevated levels of high-risk ceramides, a Mediterranean diet may be favorable to reduce the cardiovascular event risk^40^. Interestingly, higher levels of PC(16:0/22:5) seemed to be protective for cardiovascular death in patients with CAD which is in line with our results^15^. PC(16:0/22:5) belongs to omega-3 polyunsaturated fatty acids (PUFA) and are abundantly present in fatty fish. They are suggested to reduce the risk of cardiac death potentially by lowering resting heart rate, blood pressure, plasma triglycerides and improving endothelial function^43^. Previous studies investigating omega-3 supplementation were inconsistent but two recent studies have shown a positive effect in reducing future CV event risk^41,42^. An interesting topic for future studies may be to examine whether ceramide/PC ratios could be useful in selecting patients that benefit most from dietary interventions or additional medical treatments in reducing cardiovascular risk.

Recently, randomized controlled trials showed that SGLT-2 inhibitors significantly improved cardiovascular outcome in patients with heart failure in those with and without diabetes^8,44^. An experimental study showed that empagliflozin (an SGLT-2 inhibitor) significantly reduced the ceramide content of cardiac cells of diabetic rats^45^. Moreover, empagliflozin was associated with the level of ceramidase (an enzyme in the ceramide metabolism) in a proteomics study^46^. Until date, no clinical studies have been performed on the effect of ceramide levels and SGLT-2 inhibitors. Therefore, it may be interesting for future studies to examine whether SGLT-2 inhibitors alter ceramide levels of patients with established cardiovascular disease and mediate cardiovascular risk reduction.

Due to the observational study design, our findings cannot prove causality between ceramides/PCs and CV events. Based on evidence from experimental studies we could speculate, however, that these lipids contribute to atherosclerotic plaque vulnerability. We found that lipid ratios associated with MACE Cer(d18:1/24:1)/Cer(d18:1/24:0), Cer(d18:1/18:0)/Cer(d18:1/24:0) and Cer(d18:1/16:0)/PC(16:0/22:5) were also positively associated with a more vulnerable carotid atherosclerotic plaque phenotype suggesting putative underlying pathobiological mechanisms. Experimental studies have implied that ceramides drive main atherosclerotic processes such as LDL aggregation, LDL uptake across the endothelium, foam cell formation, production of reactive oxygen species, apoptosis, endothelial dysfunction and inflammatory processes^18^. Studies on the relation of ceramides/PCs and atherosclerotic plaque composition in patients are scarce. Two studies in CAD patients have revealed that increased levels of Cer(d18:1/16:0), Cer(d18:1/18:0) and Cer(d18:1/24:1) were associated with higher coronary plaque vulnerability, characterized with a higher lipid volume, necrotic core and a thinner fibrous cap using intravascular ultrasound and optical coherence tomography^17,41^. In our study, high-risk ceramides/PCs ratios were also associated with a more vulnerable atherosclerotic plaque composed of fewer SMCs and more macrophage infiltration. Experimental data has shown that ceramides induce the activity of matrix metalloproteinases (MMPs) and proinflammatory cytokines^18^. In vitro, ceramides induced apoptosis of vascular smooth muscle cells (VSMCs)^42^. It is known that increase of MMPs and loss of VSMCs contributes to thinning of the fibrous cap and subsequent plaque disruption. Additionally, pharmacological inhibition of ceramide synthetic pathway reduced macrophage content and increased SMCs content in mice^43^. Our results further contribute to evidence of the potential role of ceramides in plaque instability.

Another possible explanation for the association between EV-derived ceramides and MACE may be that high levels of ceramides reflect a general pathological state of the endothelium. The endothelium is an important source of plasma ceramides and are implicated in NO mediated vasodilatation^44^. Moreover, it has been shown that the endothelium also generates EVs and, when activated by TNFα, EVs are generated with increased ceramide content^45^. Exact underlying mechanisms how EV-derived ceramides/PCs relate to increased risk of CV events remains to be elucidated. As the biological function of distinct ceramides and PCs in atherogenesis is still poorly understood, future studies should determine whether ceramides and PCs are real effectors or solely markers of progression of atherosclerosis and subsequent CV events. Understanding the role of ceramides/PCs and related synthesizing or degrading biosynthetic enzymes in atherosclerotic diseases may lead to new potential therapeutic targets^46^.

Reported correlations of ceramides/PCs in plasma EVs with plaque characteristics in our study do not establish the origin of EVs. Determination of the cellular origin of EVs is challenging since expression of cellular surface markers on EVs are not specific to the parent cell types^47^. It is known that EVs can be released by almost all cell types such as endothelial cells, cardiomyocytes, platelets, red- or white blood cells^20^. The role of EVs in atherothrombotic processes has been well acknowledged, although exact mechanisms in vivo are unclear^20^. Future studies should explore the cellular origin of EVs because this would provide more insight in the origin of high-risk lipids and underlying processes related to atherosclerotic events.

Potential study limitations need to be addressed. First, although previous CAD cohorts found stronger associations of ceramide ratios with CV death compared to MACE^11,12,15,17^, we were not able to perform analyses on separate outcomes due to a limited number of events. Second, during follow-up no data regarding dietary patterns, medication use or adherence were available. This may have modified the observed associations between molecular lipids and MACE. Third, although the IDI may suggest potential incremental prognostic value of Cer(d18:1/24:1)/Cer(d18:1/24:0) in TEX-EVs on top of clinical risk factors, this result needs to be interpreted with caution since the improvement was minor (3%) and no improvement in C-statistic was observed. Predictive performance of ceramides could not be compared to the performance of known biomarkers for MACE^48^, such as troponin and NT-proBNP, because these data was not available. Future studies should determine the added value of ceramides for MACE prediction in comparison with these existing biomarkers. Validation studies should use clinically relevant cut points that are useful in determining treatment strategies. Last, subgroup analyses in patients undergoing CEA for asymptomatic carotid stenosis could not be performed due to low patient numbers. Ceramides and PCs may be relevant for risk stratification in this particular subgroup in light of the ongoing debate whether or not to perform CEA, but this should be further explored in appropriately designed future studies.

Major strengths include that this is the first study investigating ceramides and PCs in CEA patients and investigating these lipids in both plasma EVs and plasma. Another strength is the prospective design with validated CV outcomes. Due to the unique design of the Athero-Express biobank we were able to concomitantly investigate CV outcomes and histological atherosclerotic plaque characteristics to generate hypotheses regarding possible biological mechanisms.

To conclude, increased levels of ceramide and PCs ratios in plasma EVs, but not in unfractionated plasma, are independently associated with increased risk of MACE after CEA. These EV-derived ceramide- and ceramide/PC ratio are therefore potential biomarkers for MACE. Ceramides/PCs ratios in EVs may be useful biomarkers for selecting high-risk patients in need for intensified secondary preventive therapy, such as add-on therapy.

## Supplementary Information

Supplementary Information.

## Abbreviations

ACS: Acute coronary syndrome (STEMI, Non-STEMI, unstable angina)
CAD: Coronary artery disease
CEA: Carotid endarterectomy
CV: Cardiovascular
EVs: Extracellular vesicles
IPH: Intraplaque haemorrhage
LDL-EV subfraction: Plasma EV-subfraction that co-precipitates with LDL
MACE: Major adverse cardiovascular events
MI: Myocardial infarction
PAD: Peripheral artery disease
PC: Phosphatidylcholine
SMCs: Smooth muscle cells
TEX-EV subfraction: Total extracellular vesicle subfraction
