# Supplementary material for: Ceramides and phospholipids in plasma extracellular vesicles are associated with high risk of major cardiovascular events after carotid endarterectomy
Source: Sci Rep. 2022 Apr 1;12:5521. doi: 10.1038/s41598-022-09225-6 (PMC8975809; doi:10.1038/s41598-022-09225-6)
Supplement: Supplementary file 1 — Supplementary Information. [file 41598_2022_9225_MOESM1_ESM.docx]

**Online Supplemental**

**“Timmerman N, et al. Ceramides and ﻿phospholipids in plasma extracellular vesicles are associated with high risk of major cardiovascular events after carotid endarterectomy”**

Table of Contents

[Supplemental Materials 2](#_Toc60145894)

[**1.** **Isolation of plasma extracellular vesicles subfractions** 2](#_Toc60145895)

[**2.** **Characterization of plasma extracellular vesicles subfractions** 3](#_Toc60145896)

[**3.** **Quantification of ceramides and PCs concentrations by LC-MS/MS analysis** 3](#_Toc60145897)

[**4.** **Histological atherosclerotic plaque characterization** 4](#_Toc60145898)

[**5.** **References** 5](#_Toc60145899)

[Supplemental Tables 6](#_Toc60145900)

## **Supplemental Materials**

### **Isolation of plasma extracellular vesicles subfractions**

Plasma samples were preoperatively collected in citrate tubes and stored in -80°C until further use. For the current study, plasma samples were thawed and ceramides and phosphatidylcholines (PCs) levels were measured in unfractionated plasma and two subfractions of plasma extracellular vesicles (EVs), the LDL-EV subfraction and the TEX-EV subfraction. The protocol for isolation of the plasma extracellular vesicle (EV) subfractions was identical to the previous published study by Dekker M, et al.^1^ In short, EVs in the LDL-subfraction were precipitated using a solution of Dextran Sulphate (DS) 0.05% (MP Biomedicals), and Manganese (II) Chloride (MnCl2) 0.05M (Sigma-Aldrich). EVs in the TEX-subfraction were precipitated with Xtractt buffer (1:4, Cavadis BV). The isolation procedure for EVs is illustrated in Figure S1.

For the LDL-EV subfraction, 50uL thawed plasma sample was needed. 55uL of phosphate buffered saline (PBS, Gibco) and 5µL of magnetic dextran nanoparticles (Nanomag®-D plain, 130nm, 1:25, Micromod) were added to the sample. A solution of DS 0.05% and MnCl_2_ 0.05 M was added to a total volume of 125 μL. The sample was mixed and incubated for 5 min at room temperature. Next, the sample was placed on a bio-plex handheld magnet (Bio-Rad) for 15 minutes at room temperature to isolate the EVs. The pellet then contained the LDL-EV subfraction and LDL particles. The pellet was lysed with 125µL complete lysis-M including protease inhibitors (Roche). Magnetic nanoparticles and debris were separated from the pellet using centrifugation (10 minutes, 3200xg) and removed.

For the TEX-EV subfraction, 25 uL thawed plasma was needed. This was diluted with 80uL PBS (Gibco). 5µL of magnetic dextran nanoparticles (Nano-mag®-D PEG-OH, 130nm, 1:25, Micromod) was added. Xtractt buffer (1:4) was added to obtain a total volume of 125 μL. The remaining steps were identical to the protocol of LDL-EV subfraction isolation as described above. The remaining pellet contains EVs referred as the TEX-EV subfraction.


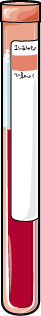

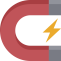


+ DS + MnCl2

& hold to magnet

Collect plasma

+ Magnetic Beads

+Xtractt ™

& hold to magnet


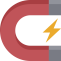


+ Lysis

+ Lysis

**EVs in LDL subfraction**

**& LDL particles**

**EVs in TEX subfraction**

Quantitative analysis on

liquid chromatography-mass spectrometry

Collect plasma

+ Magnetic Beads

Quantitative analysis on

liquid chromatography-mass spectrometry

**Figure S1.** Overview of the isolation procedure of plasma EVs.

### **Characterization of plasma extracellular vesicles subfractions**

EV characterization in the plasma EV subfractions has been reported previously*.*^1–3^ Previous data can be found in the EV-track ID (EV200044). For this study, we performed additional experiments in order to confirm the presence of ceramides/PCs in EVs. Density gradient centrifugation of the LDL EV subfraction resulted in 10 density gradient fractions. Previous studies using CD9 (EV specific antibody) western blot analysis and electron microscopy showed that vesicles are present in fractions with densities between 1.02 and 1.08 (see Figure S2, and Zhang et al, Table S4 with Figure S4)^2^ Measurement of ceramides/PCs concentrations in the 10 density gradient fractions showed peak concentrations in fractions with density 1.02-1.04 (Supplemental table S1 and Figure S3) indicating the expression of ceramides/PCs in EVs.


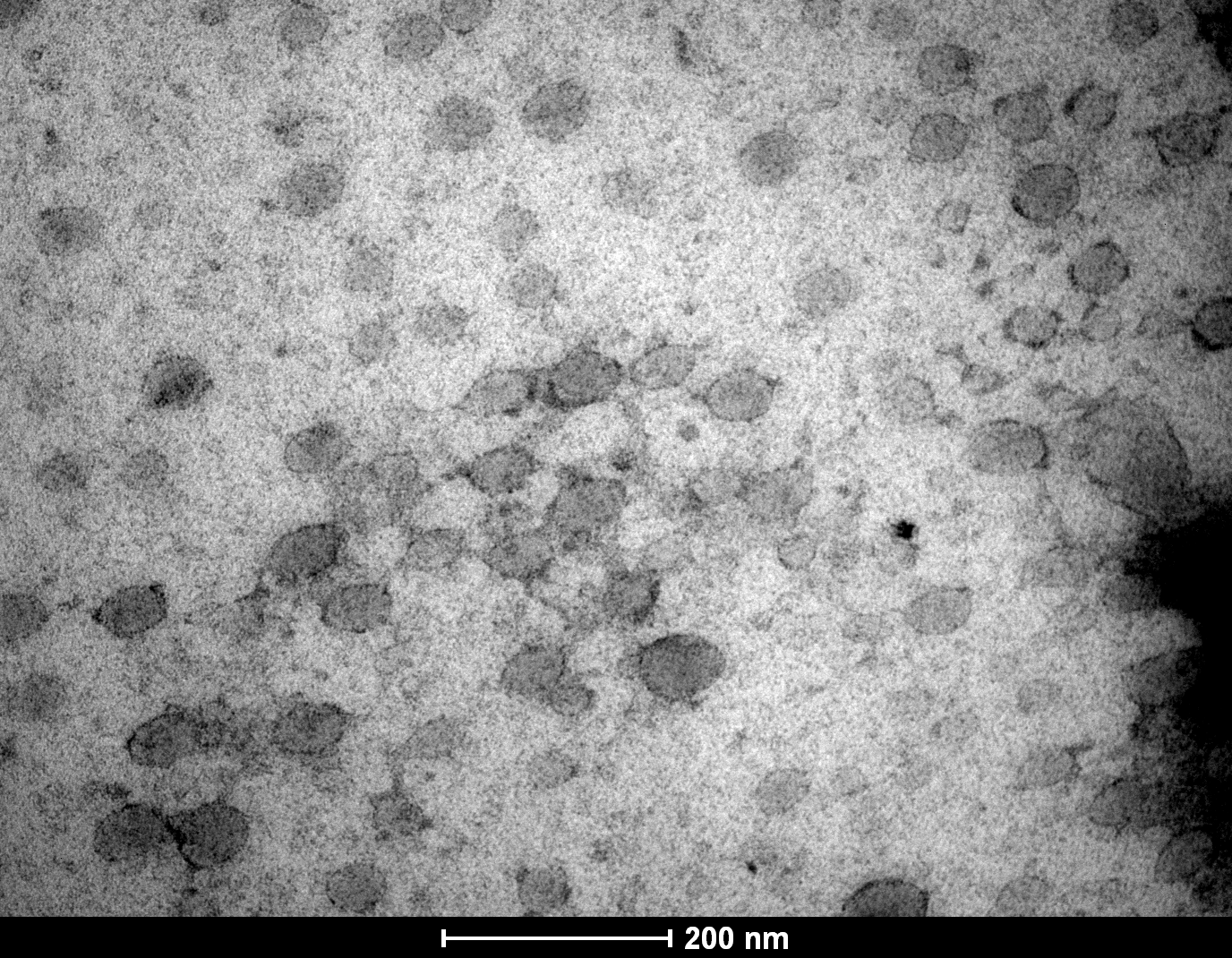

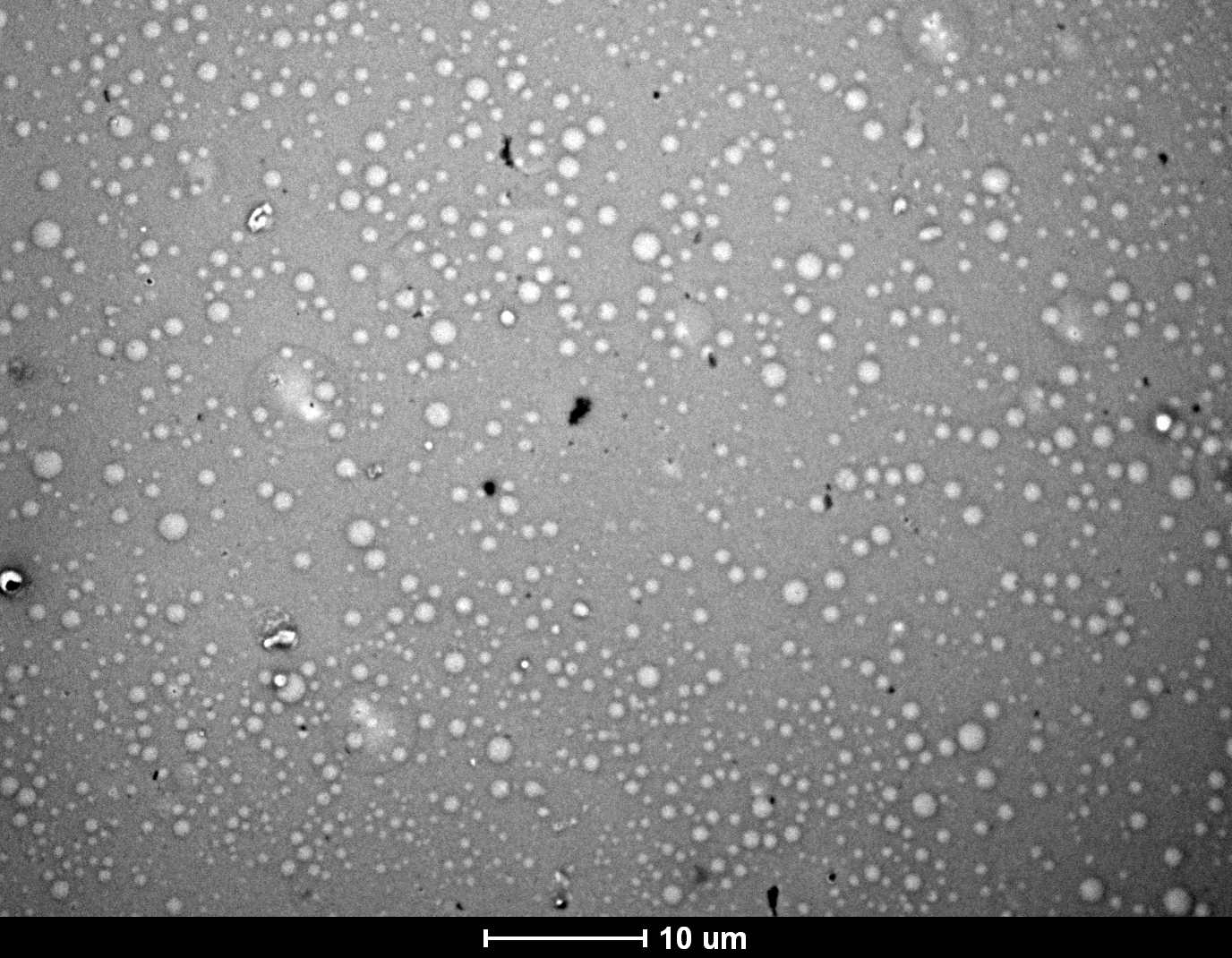

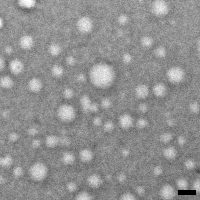

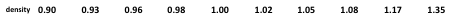

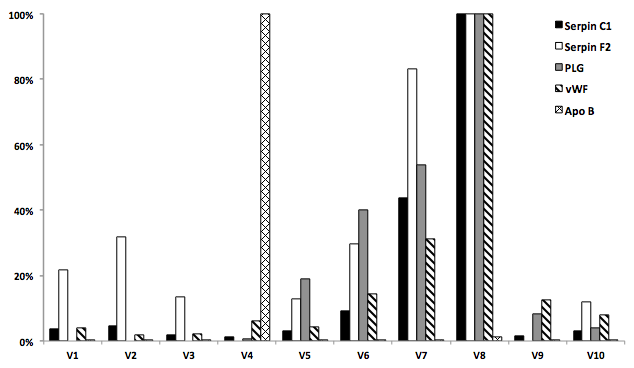

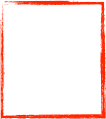

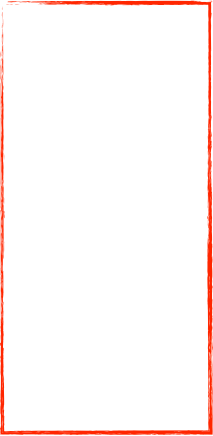

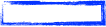

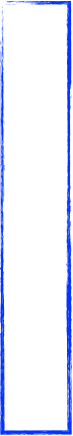


**1** Density gradient

centrifugation with

4 EV proteins and

ApoE protein levels

determined in each

of the fractions

**2** CD9 western blotting

on each of the density

gradient centrifugation

fractions

**3** Electron microscopy

on fraction V4 (ApoB) and

fraction V7 (EV proteins & CD9)

of the density gradient

centrifugation fractions


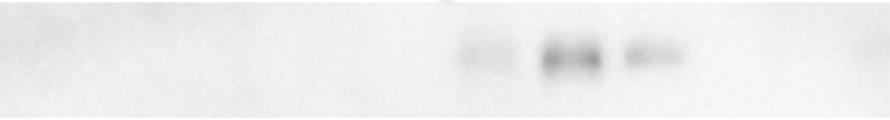


**Figure S2.** Overview of results from experiments of Zhang et al. showing that EVs are in the density gradients of 1.02, 1.05 and 1.08. Figures copied with permission from Zhang et al.^2^

Figure S2. Results from experiments of Zhang et al. indicating that EVs are in the density gradients of 1.02, 1.05 and 1.08. Figures copied with permission from Zhang et al.


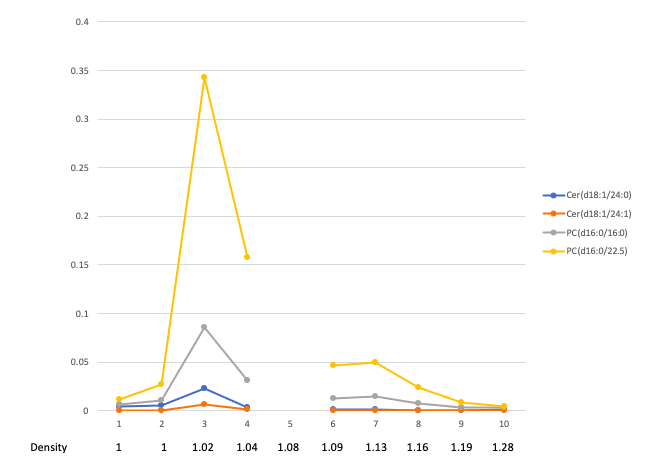


**Figure S3.** Ceramides and PCs measured in 10 density gradient fractions of the LDL-EV subfraction. Peak concentrations were observed in fractions with density 1.02-1.04. Measurements of ceramides and PCs failed in fraction with density 1.08.

| Density gradient fraction | Density | Cer  (d18:1/24:0) | Cer  (d18:1/24:1) | PC  (d16:0/16:0) | PC  (d16:0/22:5) | CD9 Western blot ^2^ |
| --- | --- | --- | --- | --- | --- | --- |
| 1 | 1 | 0.004274 | 0.000494 | 0.005988 | 0.01104 | - |
| 2 | 1 | 0.00586 | 0.000225 | 0.010485 | 0.02719 | - |
| **3** | **1.02** | **0.022336** | **0.005982** | **0.08526** | **0.34304** | **+** |
| **4** | **1.04** | **0.003636** | **0.00088** | **0.031444** | **0.15737** | **++** |
| 5 | 1.08 | NA | NA | NA | NA | **+** |
| 6 | 1.09 | 0.001024 | 0.000626 | 0.01249 | 0.046706 | - |
| 7 | 1.13 | 0.001584 | 0.000568 | 0.014656 | 0.050032 | - |
| 8 | 1.16 | 0.000388 | 0.000052 | 0.00782 | 0.02436 | - |
| 9 | 1.19 | 0.000692 | 0.000104 | 0.00328 | 0.008406 | - |
| 10 | 1.28 | 0.000804 | 0 | 0.003766 | 0.004102 | - |
| **Table S1.** Density gradient centrifugation of the LDL subfraction. Mass spectrometry (MS) shows that high levels of ceramides (Cer) and phosphatidylcholines (PC) are present in fractions 3 and 4 with densities that are positive for the Extracellular Vesicle marker CD9 in the LDL subfraction (Zhang et al 2016, S4 Fig ^2^). This shows that ceramides and PCs are present in Extracellular Vesicles. Fraction 5 was lost for MS analysis. | | | | | | |

### **Quantification of ceramides and PCs concentrations by LC-MS/MS analysis**

Ceramides and PCs concentrations were quantified in plasma, LDL-EV and TEX-EV subfractions using liquid chromatography-mass spectrometry (LC-MS/MS analysis). Ceramides and PCs concentrations were collected from the sample by extracting lipids from the sample matrix according to previously published methods.^4,5^ LC-MS/MS analysis was conducted on a Sciex TripleQuad 5500 mass spectrometer coupled to Sciex MPX LC system. Electrospray ionization in positive ion mode was used with multiple reaction monitoring. Instrument and data acquisition were controlled using Analyst® (version 1.7). The following settings were applied to all compounds in the analysis: Curtain gas, 35; ion spray voltage, 5000V; temperature, 300°C; gas 1 and gas 2, 50; declustering potential, 30; entrance potential, 10; collision exit potential, 20. Collision energy was set separately to each lipid. Chromatographic separation was performed on an Acquity BEH C18 2.1x75 mm id.1.7 µm column. Temperature was set to 60°C. Mobile phases consisted of (A) 10 mM Ammonium acetate with 0.1% formic acid and (B) 10 mM Ammonium acetate in acetonitrile:2-propanol (4:3, v/v) with 0.1% formic acid. Loading pump solvent in MPX consisted of A:B (21:79%).

Injection volume was 3 µl and flow rate was 500 µl/min. The following gradient was applied: A/B (22/78%) from 0 to 1.5 min, then B to 85% at 2 min and to 100% at 2.5 min. B was held at 100% from 2.5 min to 4.0 min, and then dropped to 78% at 4.1 min and held until 4.6 min. Both streams had the same parameters. MS analysis was performed from 1.1 min to 3.6 min which allowed multiplex to run a sample every 2.5 min. Each 96 well plate had standard line (6 points), QC samples (6) and blank samples to ensure analytical quality. Standards and QC samples were extracted the same way as the study samples. Analytical method was validated according to FDA guideline for biological sample analyses. The final concentrations of ceramides and PCs were expressed in µM.

### **Histological atherosclerotic plaque characterization**

Histological examination of the carotid atherosclerotic plaque was performed according to the standardized Athero-Express biobank protocol. Details have been published previously.^6,7^ The carotid plaque is cross-sectionally cut into segments of 5mm. The segment with the largest plaque volume was considered as the culprit lesion and allocated to immunohistochemical analysis. Semiquantitative analyses of plaque characteristics were performed on microscope with 40x magnification and expressed as no/minor or moderate/heavy staining according to predefined criteria.^6^ Hematoxylin-Eosin (H&E) staining was used for a general overview and calcifications, picrosirius red and elastin von Gieson staining for collagen, alpha-actin staining for SMCs and CD68 for macrophages. IPH was defined as hemorrhage within the plaque tissue or hemorrhage at the luminal border of the plaque as a result of plaque disruption. IPH was assessed using H&E and fibrin staining (﻿Mallory’s phosphotungstic acid-hematoxylin) and scored as present or absent. The size of the lipid core was visually estimated relative to the total plaque area (expressed as <10%, 10-40%, >40% of the total plaque area) on H&E and picrosirius red stains. In addition, SMCs, macrophages and intraplaque vessels (using CD34-antibodies) were quantified by computerized analysis software (AnalySIS 3.2, Soft Imaging Systems GmbH, Munster, Germany). SMCs and macrophage infiltration were expressed as the percentage of positive staining of the total plaque area. CD34 positive intraplaque vessels were counted in three hotspots with highest vessel density and the average number﻿ per square millimeter was calculated, as described previously.^8^ All plaques were scored by two experts who were blinded from clinical data. Substantial to perfect intraobserver- and interobserver reproducibility (_K_= 0.6-0.9) have been confirmed.^9^

### **References**

1. Dekker, M. *et al.* Plasma extracellular vesicle proteins are associated with stress-induced myocardial ischemia in women presenting with chest pain. *Sci. Rep.* **10**, 12257 (2020).

2. Zhang, Y.-N. *et al.* Extracellular Vesicle Proteins Associated with Systemic Vascular Events Correlate with Heart Failure: An Observational Study in a Dyspnoea Cohort. *PLoS One* **11**, e0148073 (2016).

3. Wang, J.-W. *et al.* Lowering Low-Density Lipoprotein Particles in Plasma Using Dextran Sulphate Co-Precipitates Procoagulant Extracellular Vesicles. *Int. J. Mol. Sci.* **19**, 94 (2017).

4. Hilvo, M. *et al.* Development and validation of a ceramide- and phospholipid-based cardiovascular risk estimation score for coronary artery disease patients. *Eur. Heart J.* **41**, 371–380 (2019).

5. Kauhanen, D. *et al.* Development and validation of a high-throughput LC–MS/MS assay for routine measurement of molecular ceramides. *Anal. Bioanal. Chem.* **408**, 3475–3483 (2016).

6. Hellings, W. E. *et al.* Composition of Carotid Atherosclerotic Plaque Is Associated With Cardiovascular Outcome. *Circulation* **121**, 1941–1950 (2010).

7. Verhoeven, B. A. N. *et al.* Athero-express: differential atherosclerotic plaque expression of mRNA and protein in relation to cardiovascular events and patient characteristics. Rationale and design. *Eur. J. Epidemiol.* **19**, 1127–33 (2004).

8. Derksen, W. J. M. *et al.* Different stages of intraplaque hemorrhage are associated with different plaque phenotypes: A large histopathological study in 794 carotid and 276 femoral endarterectomy specimens. *Atherosclerosis* **218**, 369–377 (2011).

9. Hellings, W. E. *et al.* Intraobserver and interobserver variability and spatial differences in histologic examination of carotid endarterectomy specimens. *J. Vasc. Surg.* **46**, 1147–1154 (2007).

## **Supplemental Tables**

| **Table S2. Percentages of missing covariates** | |
| --- | --- |
|  | **Missing (%)** |
| Age | 0.0 |
| Male | 0.0 |
| Current smoking | 1.0 |
| LDL-C | 4.2 |
| HDL-C | 3.4 |
| History of CAD or PAD | 0.5 |
| Cerebrovascular symptoms | 0.0 |
| Lipid lowering drug use | 0.2 |
| *CAD,* coronary artery disease; *PAD,* peripheral artery disease; *LDL-C,* Low-density lipoprotein cholesterol; *HDL-C,* high-density lipoprotein cholesterol. | |

| **Table S3. Median and interquartile ranges of ceramide/PC levels and the ratios in plasma and in the LDL- and TEX-EV subfractions** | | | | | |
| --- | --- | --- | --- | --- | --- |
| **Biomarker** |  | **No MACE (n=735)** | **MACE (n=138)** | ***p-*value** | **Missing (%)** |
| Cer(d18:1/16:0) | plasma | 0.200 [0.168, 0.238] | 0.198 [0.170, 0.238] | 0.902 | 5.5 |
|  | LDL-EV | 0.012 [0.010, 0.014] | 0.013 [0.010, 0.015] | 0.125 | 5.5 |
|  | TEX-EV | 0.009 [0.007, 0.012] | 0.009 [0.007, 0.011] | 0.523 | 1.8 |
| Cer(d18:1/18:0) | plasma | 0.075 [0.060, 0.094] | 0.076 [0.059, 0.099] | 0.746 | 5.7 |
|  | LDL-EV | 0.004 [0.003, 0.005] | 0.004 [0.003, 0.005] | 0.953 | 5.7 |
|  | TEX-EV | 0.003 [0.002, 0.005] | 0.003 [0.002, 0.005] | 0.560 | 2.5 |
| Cer(d18:1/24:0) | plasma | 2.208 [1.736, 2.683] | 2.071 [1.616, 2.522] | **0.025** | 5.2 |
|  | LDL-EV | 0.073 [0.060, 0.091] | 0.070 [0.059, 0.085] | 0.252 | 4.9 |
|  | TEX-EV | 0.115 [0.086, 0.157] | 0.096 [0.073, 0.134] | **<0.001** | 3.1 |
| Cer(d18:1/24:1) | plasma | 0.891 [0.755, 1.054] | 0.887 [0.754, 1.083] | 0.911 | 5.5 |
|  | LDL-EV | 0.043 [0.035, 0.053] | 0.046 [0.039, 0.058] | 0.056 | 5.7 |
|  | TEX-EV | 0.044 [0.033, 0.057] | 0.044 [0.031, 0.059] | 0.713 | 2.9 |
| PC(14:0/22:6) | plasma | 0.411 [0.285, 0.565] | 0.341 [0.272, 0.488] | **0.021** | 5.4 |
|  | LDL-EV | 0.034 [0.023, 0.048] | 0.029 [0.020, 0.043] | **0.047** | 5.5 |
|  | TEX-EV | 0.010 [0.006, 0.017] | 0.008 [0.004, 0.015] | **0.033** | 8.7 |
| PC(16:0/16:0) | Plasma | 9.073 [7.590, 11.04] | 9.295 [7.630, 11.08] | 0.914 | 5.2 |
|  | LDL-EV | 7.902 [6.913, 9.197] | 7.984 [6.955, 9.280] | 0.564 | 5.3 |
|  | TEX-EV | 24.66 [21.54, 27.80] | 24.39 [20.84, 27.02] | 0.140 | 2.1 |
| PC(16:0/22:5) | plasma | 31.86 [26.14, 39.14] | 29.36 [23.93, 38.37] | **0.018** | 5.0 |
|  | LDL-EV | 2.706 [2.090, 3.374] | 2.520 [1.998, 3.269] | 0.174 | 5.2 |
|  | TEX-EV | 0.933 [0.690, 1.260] | 0.863 [0.627, 1.119] | **0.010** | 3.4 |
| Cer(d18:1/16:0)/Cer(d18:1/24:0) | plasma | 0.091 [0.078, 0.107] | 0.094 [0.079, 0.111] | 0.081 | 5.5 |
|  | LDL-EV | 0.162 [0.133, 0.196] | 0.170 [0.136, 0.203] | 0.147 | 5.8 |
|  | TEX-EV | 0.075 [0.062, 0.091] | 0.084 [0.067, 0.107] | **<0.001** | 2.4 |
| Cer(d18:1/18:0)/Cer(d18:1/24:0) | plasma | 0.034 [0.028, 0.043] | 0.037 [0.028, 0.046] | 0.120 | 5.7 |
|  | LDL-EV | 0.052 [0.040, 0.067] | 0.054 [0.039, 0.070] | 0.737 | 5.6 |
|  | TEX-EV | 0.028 [0.021, 0.037] | 0.032 [0.022, 0.046] | **0.022** | 2.7 |
| Cer(d18:1/24:1)/Cer(d18:1/24:0) | plasma | 0.410 [0.339, 0.490] | 0.420 [0.365, 0.518] | 0.070 | 5.4 |
|  | LDL-EV | 0.589 [0.480, 0.722] | 0.646 [0.511, 0.749] | **0.049** | 5.2 |
|  | TEX-EV | 0.379 [0.303, 0.463] | 0.421 [0.330, 0.524] | **<0.001** | 2.3 |
| Cer(d18:1/18:0)/Cer(d18:1/16:0) | plasma | 0.377 [0.322, 0.449] | 0.372 [0.321, 0.458] | 0.973 | 5.3 |
|  | LDL-EV | 0.323 [0.267, 0.390] | 0.300 [0.252, 0.388] | 0.345 | 5.2 |
|  | TEX-EV | 0.381 [0.298, 0.472] | 0.379 [0.291, 0.476] | 0.845 | 1.7 |
| Cer(d18:1/16:0)/PC(16:0/22:5) | plasma | 0.006 [0.005, 0.008] | 0.007 [0.006, 0.008] | **0.012** | 5.6 |
|  | LDL-EV | 0.005 [0.004, 0.005] | 0.005 [0.004, 0.006] | **0.002** | 5.5 |
|  | TEX-EV | 0.010 [0.007, 0.013] | 0.011 [0.008, 0.015] | **0.005** | 1.8 |
| Cer(d18:1/18:0)/PC(14:0/22:6) | plasma | 0.182 [0.122, 0.271] | 0.205 [0.135, 0.336] | **0.035** | 5.8 |
|  | LDL-EV | 0.111 [0.078, 0.168] | 0.124 [0.080, 0.205] | 0.116 | 6.1 |
|  | TEX-EV | 0.329 [0.190, 0.595] | 0.386 [0.207, 0.716] | 0.193 | 8.5 |
| Values are displayed as median [interquartile] concentrations in µM. *P-*values originate from comparison of median levels by Mann-Whitney U test between patients that experienced MACE versus those who did not. Values in bold indicate p<0.05. Missing measurements of ceramides/PCs were randomly divided across all samples and were due to technical failures. Cer, ceramide; PC, phosphatidylcholine. LDL-EV indicates the LDL-EV subfraction, TEX-EV indicates the TEX-EV subfraction. | | | | | |

| **Table S4. Subanalyses corrected for statin use.** | | | |
| --- | --- | --- | --- |
| **Biomarker** |  | **HR (95% CI)** | ***p-*value** |
| Cer(d18:1/16:0) | plasma | 0.98 (0.77-1.23) | 0.836 |
|  | LDL-EV | 1.22 (0.98-1.51) | 0.069 |
|  | TEX-EV | 0.94 (0.56-1.57) | 0.804 |
| Cer(d18:1/18:0) | plasma | 1.00 (0.80-1.24) | 0.985 |
|  | LDL-EV | 1.06 (0.86-1.30) | 0.583 |
|  | TEX-EV | 0.93 (0.68-1.26) | 0.618 |
| Cer(d18:1/24:0) | plasma | 0.86 (0.68-1.07) | 0.178 |
|  | LDL-EV | 1.05 (0.86-1.28) | 0.648 |
|  | TEX-EV | 0.73 (0.53-1.02) | 0.063 |
| Cer(d18:1/24:1) | plasma | 0.94 (0.76-1.18) | 0.607 |
|  | LDL-EV | 1.23 (1.00-1.52) | **0.046** |
|  | TEX-EV | 0.92 (0.69-1.23) | 0.588 |
| PC(14:0/22:6) | plasma | 0.88 (0.70-1.10) | 0.263 |
|  | LDL-EV | 0.94 (0.76-1.16) | 0.557 |
|  | TEX-EV | 0.89 (0.71-1.12) | 0.326 |
| PC(16:0/16:0) | plasma | 1.09 (0.86-1.37) | 0.481 |
|  | LDL-EV | 1.17 (0.94-1.46) | 0.149 |
|  | TEX-EV | 0.94 (0.76-1.15) | 0.540 |
| PC(16:0/22:5) | plasma | 0.94 (0.76-1.15) | 0.532 |
|  | LDL-EV | 0.98 (0.80-1.20) | 0.867 |
|  | TEX-EV | 0.85 (0.67-1.07) | 0.166 |
| Cer(d18:1/16:0)/Cer(d18:1/24:0) | plasma | 1.09 (0.86-1.37) | 0.485 |
|  | LDL-EV | 1.06 (0.85-1.33) | 0.592 |
|  | TEX-EV | 1.31(1.03-1.67) | **0.026** |
| Cer(d18:1/18:0)/Cer(d18:1/24:0) | plasma | 1.08 (0.87-1.34) | 0.470 |
|  | LDL-EV | 0.90 (0.72-1.12) | 0.349 |
|  | TEX-EV | 1.22 (1.00-1.50) | 0.052 |
| Cer(d18:1/24:1)/Cer(d18:1/24:0) | plasma | 1.07 (0.86-1.32) | 0.558 |
|  | LDL-EV | 1.06 (0.86-1.30) | 0.616 |
|  | TEX-EV | 1.29 (1.07-1.56) | **0.008** |
| Cer(d18:1/18:0)/Cer(d18:1/16:0) | plasma | 0.96 (0.79-1.16) | 0.657 |
|  | LDL-EV | 0.90 (0.74-1.09) | 0.269 |
|  | TEX-EV | 0.93 (0.78-1.11) | 0.427 |
| Cer(d18:1/16:0)/PC(16:0/22:5) | plasma | 1.20 (0.97-1.49) | 0.086 |
|  | LDL-EV | 1.24 (1.03-1.50) | **0.025** |
|  | TEX-EV | 1.25 (0.88-1.77) | 0.220 |
| Cer(d18:1/18:0)/PC(14:0/22:6) | plasma | 1.10 (0.86-1.41) | 0.453 |
|  | LDL-EV | 1.86 (0.35-9.99) | 0.467 |
|  | TEX-EV | 0.80 (0.37-1.73) | 0.569 |
| Multivariable analyses were corrected for statin use, LDL-cholesterol and HDL-cholesterol, age, history of coronary artery disease, history of peripheral artery disease, cerebrovascular symptoms and current smoking.  HR indicates the hazard ratio for the 3-year postoperative risk of MACE per one standard deviation increase in concentration of ceramides, PC or ratio either in plasma or plasma extracellular vesicles (EVs). Values in bold indicate p<0.05. CI, confidence interval. Cer, ceramides; PC, phosphatidylcholine. LDL-EV indicates LDL-EV subfraction, TEX-EV indicates TEX-EV subfraction | | | |

**Supplemental data Table S5**

| **Lipid class** | **Lipid name** | **n** | **n_flags** | **rsd %** | **mean** | **sd** |
| --- | --- | --- | --- | --- | --- | --- |
| Cer d18:1 | Cer(d18:1/16:0) | 258 | 0 | 11.59 | 0.199 | 0.023 |
| Cer d18:1 | Cer(d18:1/18:0) | 258 | 0 | 11.55 | 0.073 | 0.008 |
| Cer d18:1 | Cer(d18:1/24:0) | 258 | 0 | 12.25 | 2.063 | 0.253 |
| Cer d18:1 | Cer(d18:1/24:1) | 258 | 0 | 12.22 | 1.278 | 0.156 |
| PC | PC(d14:0/22:6) | 258 | 0 | 10.79 | 0.704 | 0.076 |
| PC | PC(d16:0/16:0) | 258 | 0 | 11.42 | 8.882 | 1.015 |
| PC | PC(d16:0/22:5) | 258 | 0 | 10.84 | 33.702 | 3.655 |
| **Table S6** Calculation of Relative Standard Deviation using 6 Quality Control samples per plate. At least 5 out of 6 QC per plate should be below 15% and the overall RSD (including samples >15%) should be below 15%. N= total number of QC samples added in this analysis. | | | | | | |

## **Supplemental Figures**

**Supplemental Figure S4.**

Calibration plots of the A) clinical model and B) clinical model including the biomarker Cer(d18:1/24:1)/Cer(d18:1/24:0) in TEX-EVs


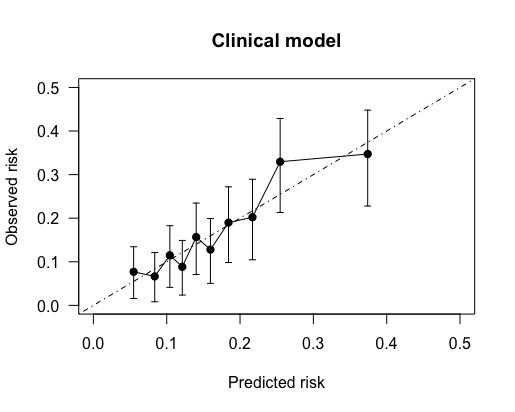


1. The clinical model including age, history of CAD and/or PAD, cerebrovascular symptoms, current smoking, LDL-C and HDL-C

**
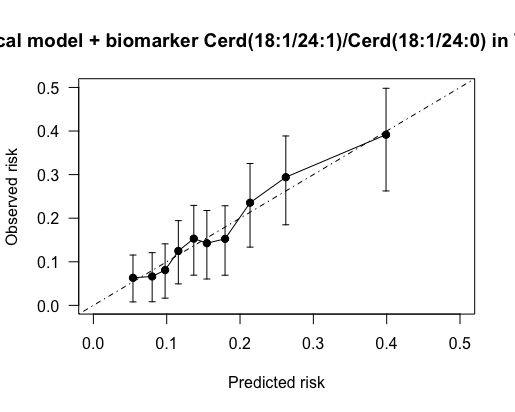
**

1. Clinical model + the biomarker Cer(d18:1/24:1)/Cer(d18:1/24:0) in TEX-EVs
